# The early life education of the immune system: Moms, microbes and (missed) opportunities

**DOI:** 10.1080/19490976.2020.1824564

**Published:** 2020-10-12

**Authors:** Nitya Jain

**Affiliations:** Mucosal Immunology and Biology Research Center, Massachusetts General Hospital for Children, Charlestown, MA, USA

**Keywords:** Perinatal immune system, window of opportunity, layered immunity, microbiota, immune education

## Abstract

The early life immune system is characterized by unique developmental milestones. Functionally diverse immune cells arise from distinct waves of hematopoietic stem cells, a phenomenon referred to as ‘layered’ immunity. This stratified development of immune cells extends to lineages of both innate and adaptive cells. The defined time window for the development of these immune cells lends itself to the influence of specific exposures typical of the early life period. The perinatal immune system develops in a relatively sterile fetal environment but emerges into one filled with a multitude of antigenic encounters. A major burden of this comes in the form of the microbiota that is being newly established at mucosal surfaces of the newborn. Accumulating evidence suggests that early life microbial exposures, including those arising *in utero*, can imprint long-lasting changes in the offspring’s immune system and determine disease risk throughout life. In this review, I highlight unique features of early life immunity and explore the role of intestinal bacteria in educating the developing immune system.

At the time of writing this review, the world was coming to grips with the scale of the COVID-19 pandemic caused by the novel coronavirus, SARS-CoV-2. The virus had infected millions of people worldwide and claimed hundreds of thousands of lives. Epidemiological data indicated, however, that the disease affected far fewer children than adults. Children and infants that did test positive for SARS-CoV-2 were largely asymptomatic or experienced a mild form of the disease. Children are usually more susceptible to respiratory viral infections and not less prone to developing severe acute respiratory distress syndrome (ARDS), a grim presentation of COVID-19. As demonstrated in the H1N1 flu pandemic of 2009, being under the age of 1 year was a significant risk factor for ARDS and the more severe form of the disease.^1^ Numerous hypotheses have been proposed for the mild COVID-19 disease in children including unique characteristics of the early life immune system, circulating maternal antibodies, and protective memory T and B cells from prior coronavirus infections that may limit damage from SARS-CoV-2 infection.^2^ Newer reports also indicate that some children infected with SARS-CoV-2 may progress to a multisystem inflammatory syndrome (MISC) resembling Kawasaki disease.^3^ The pathophysiology is yet unknown and this appears to be a rare complication affecting some children over others. The bottom line is that many infectious diseases affect children differently than adults. Understanding these differences, including how the immune system develops and the environmental and host factors that dictate plasticity in early life immune responses, will yield important insight into disease pathogenesis and inform development of new therapeutics.

The early life represents a period of unique immune development during which the foundation for lifelong immunity is laid. Epidemiological data link pre- and post-natal encounters with susceptibility to chronic inflammatory and metabolic diseases in later life.^4–6^ Distinct immune cell subsets arise in early life that are wired to function in an environment with unique exposures. One exposure of critical importance is the burgeoning intestinal microbiota, the collection of diverse bacteria, archaea, viruses, and fungi, that regulate aspects of immune system development and function. Here, I review the premise for an early life time window when microbial encounters influence the development of select immune phenotypes.

## Early life window of opportunity

The Developmental Origins of Health and Disease (DOHaD) hypothesis proposes that exposure to certain environmental stimuli during critical periods of prenatal development have significant consequences on an individual’s short- and long-term health.^7^ The developing fetus may respond to hostile uterine environments, resulting, for example, from poor maternal nutrition or infection, by changing endocrine and metabolic activity that slow down its growth rate. Resulting irreversible changes in the structure and function of vital organs are exacerbated by subsequent environmental exposures during infancy and childhood that condition the risk of disease in later life. A critical foundation of the DOHaD hypothesis is the concept of developmental plasticity, or the ability of an organism to develop in diverse ways, depending on the particular environment and setting.^8^ The immune system displays considerable developmental plasticity. Hematopoietic stem cells (HSCs) that give rise to numerous blood and immune cells are able to sense diverse stimuli to influence the type and function of cell lineages generated.^9–11^ Plasticity is also observed during the generation of antigen-specific immune responses, the quality of which are determined by numerous macro- and micro-environmental factors that regulate the differentiation of specific effector cells.^12,13^ Thus, the principles of the DOHaD hypothesis are applicable to the immune system too, and numerous studies have established links between maternal nutritional or infectious status and the offspring’s immunity and subsequent disease risk.^14,15^ For example, maternal malnutrition results in decreased placental transfer of maternal antibody to the fetus. This has long-lasting effects on B cell development in offspring that correlates with susceptibility to allergies and autoimmune diseases.^16,17^ Chronic helminthiasis during pregnancy promotes Th2 cytokine responses and regulatory T cell differentiation in offspring^18,19^ that correlates with diminished vaccine responses and increased susceptibility to tuberculosis.^20^

However, while unfavorable early life exposures certainly increase the risk of disease in offspring, nonpathogenic microbial exposures may in fact promote the generation of protective immunity. This idea was first proposed by Strachan in 1989 to explain the inverse correlation between birth order and incidence of allergic diseases in families in Britain.^21^ Shared living spaces and air was presumed to increase microbial spread in younger children of large families, which favored the development of protective immunity to hay fever in younger siblings. Similar observations have since been made for a variety of environmental conditions that promote microbial exposure early in life. The prevalence of atopy was found to be lower in children who started to attend day care at a younger age (6–11 months) compared to those who started at an older age.^22,23^ Farm living is associated with exposure to higher diversity of bacteria and fungi that appear to protect from atopy.^24^ Conversely, higher incidences of asthma and hypersensitivity disorders were observed in children raised in ‘cleaner’ urban dwellings compared to rural and farm environments.^25–27^ Thus, while the DOHaD hypothesis proposes a prenatal developmental period during which adversarial exposures increase the risk of disease, the ‘*early life window of opportunity*’ argument advances the notion of a defined perinatal period during which specific microbial exposures lead to a favorable imprint on immunity that reduces risk of disease later in life. As windows of opportunity go, it is predicted that this period of immune malleability would be short, would not appear again in later life and would lead to long-term beneficial health consequences. It is of interest, therefore, to understand^1^ when the window of opportunity arises in early life,^2^ the identity of immune cells generated during this period that are likely to be receptive to microbial influence,^3^ the nature of microbes and their components that play a role in educating the early life immune system and of course,^4^ the practical implications of this phenomenon to ultimately develop strategies that promote health and prevent the incidence of chronic inflammatory diseases. I discuss these in some detail below.

## Perinatal microbial encounters

**Figure 1.**
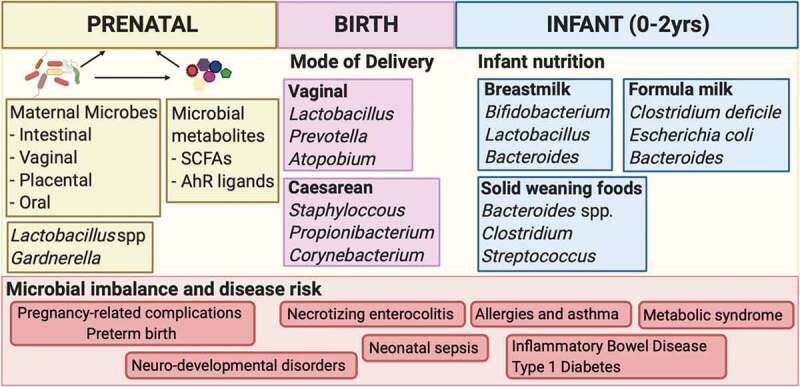
**Perinatal microbial encounters**. Three windows of influential microbial encounters may be distinguished in the pre- and post-natal period. Maternal microbes as well as their metabolites may influence the uterine environment in which the fetus develops. Delivery mode shapes the initial microbial inoculum of the newborn while nutrition drives microbial composition during the postnatal period. Microbial imbalance during these periods alter susceptibility to pathogens and inflammatory disease in early and later life.

An appreciation of the duration of the window of opportunity that establishes trajectories of disease risk begins with an understanding of when microbial exposures occur early in life. Three windows of influential microbial encounters may be distinguished in the pre- and post-natal period: microbial exposure *in utero* during pregnancy, microbes acquired at the time of delivery, and microbes established postnatally by the acquisition of maternal and environmental bacteria (Figure 1). Thus, the first 1000 days of life, that is, the period from conception to 2 years of age, may provide opportunities for microbial imprint of immunity that set thresholds of reactivity throughout life.

### Pregnancy-associated microbiota

The maternal microbiota in the gut and reproductive tract undergoes significant changes during pregnancy that is influenced by a combination of hormonal, metabolic, and immunological factors as well as by maternal diet, supplement intake, and antibiotics use.^28^ Alterations in the vaginal microbiota have been linked to preterm birth, a major cause of worldwide neonatal morbidity and mortality.^29^ Activation of maternal immunity leading to preterm birth has been well documented;^30^ however, an impact on the developing fetal immune system and subsequent long-term health consequences in offspring cannot be ruled out. Maternal gut microbes metabolize dietary components that is passed to the developing fetus across the placenta. The placenta is the highly adapted primary interface between the mother and the developing fetus that facilitates the exchange of nutrients, gases, xenobiotics, and waste products while protecting the fetus from rejection by the maternal immune system. It is also the focus of a controversial and debated topic about the existence of an intra-uterine microbiome in a healthy pregnancy. The long-held premise that the fetus develops in a sterile environment *in utero* is being challenged.^31^ Some studies have demonstrated the presence of low abundance commensal bacteria in the healthy placenta,^32,33^ while others refute this premise of a placental microbiome.^34,35^ Distinct bacterial profiles were also reported in gestational week 20 fetal intestines, findings that need to be corroborated in other independent cohorts.^36^ While exposure of the fetus to live bacteria *in utero* remains to be confirmed, the fetal tissues are likely exposed to numerous microbial metabolites and microbial fragments of maternal origin that are transferred across the placenta from the maternal serum. Gut microbes can induce an IgG antibody response in hosts that protect from systemic infections.^37^ Importantly, these commensal-specific IgG antibodies can be transferred across the placenta to the offspring where they regulate mucosal CD4^+^T cell responses to commensal antigens early after birth.^38^ Maternal IgG antibodies can also facilitate transfer of bacterial compounds themselves across the placenta.^39^ In a mouse model where bacteria were only present during pregnancy, maternal antibodies enhanced transfer of bacterial compounds, including ligands for the aryl hydrocarbon receptor (AhR), from the mother to the offspring where they primed the developing immune system. Maternal enteric microbes also ferment dietary fibers to the short-chain fatty acid (SCFA) acetate, that suppresses allergic airway disease, a mouse model for human asthma, by enhancing Treg cell numbers and function in adult offspring.^40^ In the context of a maternal infection, bacterial peptidoglycan, which is a ligand for Toll-like Receptor 2 (TLR2), traversed the placenta to influence fetal neuro-proliferation.^41^ Passage of other microbial TLR ligands across the placenta that influence fetal immune development remains a formal possibility.

### Delivery associated microbiota

Parturition is the next major point of microbial exposure for the developed fetus. The first microbial contacts of a newborn outside of the uterus are influenced by the mode of delivery and the presence of microbial communities on the maternal skin and in the reproductive tract. While the composition of the neonatal microbiota across different body sites early after birth is not impacted by the delivery mode, gut bacterial communities display significant differences in babies born by either vaginal or Cesarean-section (CS) delivery.^42–44^ Vaginally delivered newborns display early enteric communities resembling the maternal vaginal and fecal microbiota, a result reflective of their prolonged transit through the birth canal. CS babies bypass contact with maternal mucosal surfaces and their initial microbial exposure consists of maternal skin bacteria and other environmental bacteria.^45^ The use of antibiotics perinatally by mother-infant dyads significantly impacts the neonatal microbiome and likely contributes to the decreased diversity of the enteric microbiota observed in CS infants.^44,46^ Studies have attempted to establish a correlation between mode of delivery to disease susceptibility in later life. CS babies may be at higher risk for developing allergies and asthma compared to vaginally delivered babies.^47,48^ A study of a Danish register-based cohort reported CS babies to have increased susceptibility for chronic inflammatory diseases including diabetes, arthritis, inflammatory bowel disease, and celiac disease.^49^ However, another study found no link between delivery mode and risk for celiac disease.^50^ Numerous factors accompanying birth may complicate interpretation of such studies, including the use of antibiotics, dominant mode of newborn nutrition immediately after birth and maternal stress and health.^51^ Further, while it is evident that the mode of delivery affects the quality of pioneer microbes introduced into newborns after birth, whether it also impacts subsequent patterns of colonization in the first 3 months of life remains unclear.^45,52^ Irrespective of birth mode, any observed differences in the earliest microbial community structure typically disappear after 6 months of life with the diversification of the infant diet.^53,54^

### Postnatal microbiota

Nutrition, in the form of breastmilk, formula milk, and solid weaning foods, has a defining role in establishing colonization patterns of the infant gut microbiota after initial maternal inoculum at delivery.^55,56^ The TEDDY study established that breast milk consumption was the most significant factor associated with the microbiome structure early in life.^57^ Numerous epidemiologic studies have documented differences in the composition of the gut microbiota in breastfed and formula-fed infants.^58,59^ Breastmilk itself contains viable bacteria originating from the maternal gut and infant oral cavity that influences the composition of the enteric microbiome.^60^ Exclusive breast-feeding in the first 4 months of life was found to prevent atopic dermatitis (AD) in infants at high risk of atopy.^61^ However, in another study, breast-feeding for at least 3 months was not significantly protective against the development of AD compared with partial breast-feeding or use of conventional formula.^62^ In addition to nutrition, numerous environmental and host factors also contribute to the step-wise progression of the infant microbiome toward adult-like configurations that are typically reached by 2–3 years of age.^63^

## Perinatal immune development

**Figure 2.**
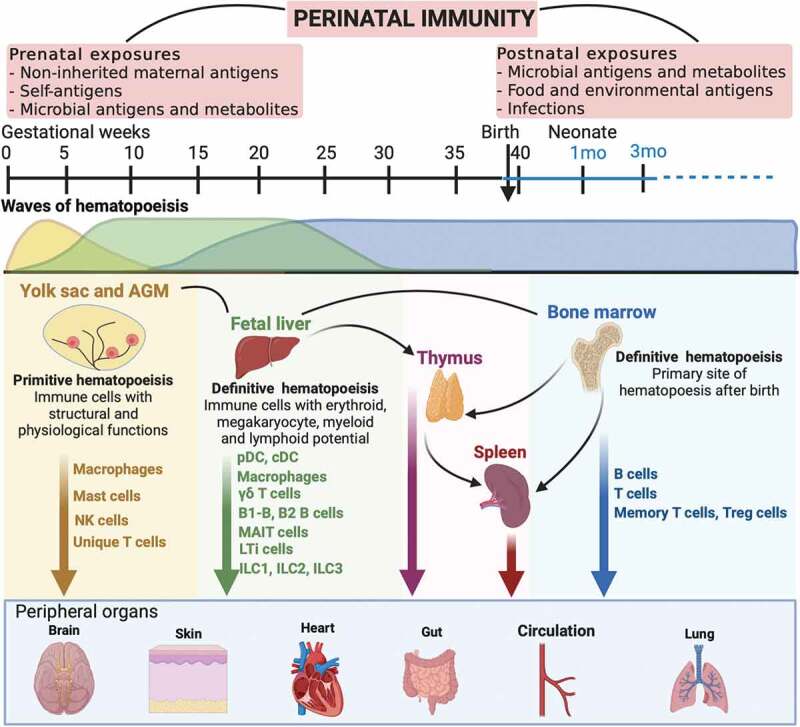
**Perinatal immune development**. Immune function in early life is shaped by specific prenatal and postnatal exposures. Waves of hematopoietic stem cells arise from the yolk sac, fetal liver and the bone marrow over gestation and generate distinct lineages of immune cells that serve the early life milieu.

The early life mammalian immune system has evolved to develop in functional layers, likely to counter an array of exposures over ontogeny.^64^ Distinct hematopoietic stem cells give rise to unique lineages of lymphoid and myeloid cells at specified times during development, creating layers of immune cell populations that collectively endow the immune system with vast capabilities (Figure 2). Immune function in the perinatal period is defined by two sets of antigenic experiences. First, the immune system must tolerate continuous exposure to an array of non-inherited maternal antigens as well as a growing repertoire of self-antigens *in utero* to ensure fetal viability during pregnancy and to avoid autoimmunity postnatally.^65^ Second, it must enable the colonization of mucosal surfaces by trillions of bacteria, a process initiated immediately after birth, to ensure the establishment of a diverse, robust microbiota that is restrained and well tolerated.^66^ Numerous components of the innate and adaptive immune systems play specialized roles in these processes and cell subsets with suppressive activity as well as potent pro-inflammatory activity may be found at various tissue sites in the fetus and in the newborn. These mechanisms establish fetal tolerance and also prepare for the transition to the postnatal milieu to jumpstart an immune program that contends with the establishment of the microbiota and encounters with a multitude of antigens. Given these specific developmental demands on perinatal immunity, it is not surprising, therefore, that susceptibility to certain infections and severe inflammatory diseases such as necrotizing enterocolitis (NEC) and neonatal sepsis are a significant threat in the early life period.^67^ This susceptibility tracks with the development of the immune system, occurring within specific time windows that parallel waves of immune cell generation and tissue colonization.^68^ For example, while immune cells migrate to the skin and gut during gestation, lung resident Th2 effector cells and ILCs are only found after birth,^69,70^ contributing to the increased susceptibility to RSV infection in newborns.^71,72^ A discussion of the perinatal generation of immune lineages follows to provide a framework for assessing impact of microbial exposures on developmental immune imprinting.

### Fetal immunity

*Yolk sac and aorta-gonad mesonephros (AGM) hematopoiesis*: Immune cells that arise from the first wave of hematopoiesis in the yolk sac have unique structural and physiological functions and equip the embryo with a repertoire of innate effectors consisting primarily of macrophages, mast cells, and Natural Killer (NK) cells. Long-lived macrophages generated from yolk-sac progenitors persist in tissues such as the liver, lung, brain, and epidermis,^73,74^ while those from HSC-derived monocytes are found in the gut, lung, and heart.^75^ Mast cells also display a similar ontogeny to macrophages and may arise from both yolk-sac and HSC-derived precursors that imprint them with distinct patterns of tissue residency.^76,77^ Pre-thymic lymphoid progenitors with long-term T cell engraftment capacity are found in the AGM region and arise before the appearance of embryonic HSCs in humans.^78–80^ In murine embryos, a subset of innate-like B cells called B-1 cells arise from hematogenic endothelia in both the yolk sac and the AGM region prior to HSCs, and seed the fetal liver.^81^

*Fetal liver hematopoiesis*: The next wave of hematopoiesis originates from the fetal liver where definitive HSCs with the full complement of erythroid, megakaryocyte, myeloid, and lymphoid potential may be found. Numerous innate and innate-like lymphocytes arise from common lymphoid progenitors in the fetal liver including innate lymphoid cells (ILCs), γδ T cells, and mucosal-associated invariant T cells (MAIT cells).^82^ While most mature ILC subsets are absent from lymphoid and non-lymphoid tissues such as the skin, kidneys, lungs, and spleen in early fetal development,^82^ ILC2s and ILC3s are present in the fetal gut.^83,84^ Antigen-presenting cells (APCs) including monocytes/macrophages, plasmacytoid dendritic cells (pDC), and conventional DC (cDC) subsets may be found in the fetal spleen, skin, thymus, and lungs by 12 weeks post-conception (PCW).^82,85^ HSC-derived innate B-1 cells in mice^81^ and adaptive B cell (B-2) precursors in humans are found in the fetal liver from 7PCW and mature B cells by 9PCW.^82^

*Bone marrow (BM) hematopoiesis*: At mid-gestation in humans and just before birth in mice, the BM becomes the primary site of hematopoiesis and the major source of B cells. B cells and follicular DCs form follicles in the fetal spleen,^86,87^ enabling antigen presentation to B cells. B cells are also found in the fetal intestine where they display phenotypes of follicular and transitional B cells but not plasma cells.^84^ While neutrophil progenitors abound in the fetal liver and BM, mature neutrophils are only found in the BM by 14 PCW where their numbers remain extremely low^88^ until immediately after birth.^89,90^

*Thymus*: The human thymus begins to develop by 8PCW and the first T cells appear in the periphery by 12–14 PCW.^91,92^ Thymus specific progenitors from the fetal liver and bone marrow seed the thymus and both γδ and αβ T cells including regulatory T (Treg) cells arise in the human fetus.^93–96^ In mice, however, except for the Vγ3/Vδ5 TCR expressing γδ T cell population that arises by embryonic day 15 (E15), most γδ and αβ T cell subsets arise in the thymus after birth in the first week of life.^65,97^ Memory T cells have also been found in the fetal spleen^98,99^ and in tissues including the skin^100^ and intestine,^101^ suggesting that complete T cell maturation can occur *in utero*. A unique feature of naïve T cells arising from fetal HSCs is their increased propensity to differentiate into Treg cells upon antigen encounter, a mechanism that likely establishes tolerance and prevents damaging alloreactivity *in uter*o.^102,103^ CD1d-restricted Vα24^+^ invariant natural killer T (iNKT) cells are found in the early fetal thymus^104^ but their numbers decline with gestational age such that they are very rare in the neonatal thymus. However, iNKT cells are highly prevalent in the small intestines as well as the lungs, spleen, and mesenteric lymph nodes during the second trimester.^105^ MR-1 restricted MAIT cells are another class of innate-like T cells that have been found in the human fetal small intestine.^106^

### Postnatal immunity

Early life represents a period of dramatic escalation in antigenic encounters by the relatively naïve and still developing newborn immune system. There is significant overlap of fetal and postnatally derived hematopoietic precursor cells in the period immediately after birth that influence the quality of immune cells generated and the overall functionality of the immune system. It is well established that newborns are highly susceptible to infections by pathogens and commensals, mostly due to insufficient, inappropriate, and biased immune responses. Dampened early life immune responses also necessitate pediatric vaccination strategies to include multi-dose immunization schedules with administration of doses later in infancy to increase immunogenicity and induce protective responses. However, overly robust immune responses are also observed in infants in developed countries, who have become increasingly sensitive to acquiring autoimmune diseases such as Type 1 Diabetes (T1D) as well as allergies and asthma. While the neonate has historically been considered to be immature in its immune characteristics, evidence clearly indicates that it is capable of mounting a range of pro- and anti-inflammatory responses.^68,107,108^

*Innate immunity*: Cells of the innate immune system are the first responders to the massive antigenic exposure that follows immediately after birth. The manner in which the innate immune system responds in early life is critical for the initiation and maintenance of host defense throughout life. The immune system recognizes microbial pathogens as well as commensal microbes via pattern-recognition receptors (PRRs) that are expressed on a variety of host cells including innate and adaptive immune cells.^109^ Appropriate regulation of these receptors is essential to prevent damaging inflammation at mucosal sites during the establishment of the commensal microbiota in early life as well as to generate productive responses against pathogenic viruses and bacteria. Failure of these mechanisms, especially in preterm infants, results in devastating disease such as neonatal sepsis and NEC as well as ineffective responses that hamper viral clearance.^68^ Decreased expression of TLR4 on monocytes from preterm and very-low birth weight infants and their subsequent lack of cytokine production has been suggested to contribute to susceptibility to infections with Gram-negative bacteria.^110,111^ The expression and cellular distribution of most other TLRs are similar between term newborn and adult monocytes.^112^ However, despite similar basal TLR expression, the functional consequences of TLR engagement in neonates are distinct. TLR stimulation of whole blood from preterm infants results in massive production of IL-10 whereas production of Th17 cell-promoting cytokines IL-6 and IL-23 dominates in term infants.^112^ pDCs are one of the major sources of Type 1 IFN following TLR triggering during viral infections and can be recruited efficiently to the respiratory tract of infants suffering from acute viral respiratory infections. However, they display dampened responses in RSV infected neonates and are defective in RIG-I dependent type 1 IFN responses.^113^ Interestingly, monocyte derived DCs as well as pDCs in human cord blood can potently produce IL-12p70 and Type 1 IFNs upon exposure to human CMV, HIV, and HSV.^114–116^ Thus, context dependent activation of TLRs on pDCs appears to impact innate immune responses to different viral triggers.

*Adaptive Immunity*: Neonatal adaptive immunity has been historically viewed as being deficient or deviant. Studies demonstrated that exposure to antigen before or during the neonatal period led to the inactivation on absence of T cells capable of mounting an immune response, a deficiency referred to as neonatal tolerance.^117,118^ Neonatal T cells were also shown to preferentially produce T helper type 2 (Th2) cytokines IL-4, IL-5, and IL-13 upon stimulation,^119,120^ a deviant response that increased susceptibility to infections and allergies during early stages of development.^121^ Accumulating evidence, however, has led to a revision of this paradigm and it is now appreciated that neonatal T cells are a distinct population of T cells with unique capacities that are well adapted to the perinatal period.^122^ Neonatal and adult T cells are derived from distinct progenitor cells that imprint in them specific functionalities; for example, a preference for Th2 and Treg cell differentiation in CD4^+^ T cells and the ability for rapid effector production in CD8^+^ T cells.^103,123,124^ Distinct gene expression profiles of neonatal T cells observed even prior to stimulation suggest that they may be more responsive upon antigen exposure.^123,125–128^ Neonatal T cells also respond to microbial ligands by virtue of their expression of numerous TLRs including TLR2 and TLR5.^129–131^ Thus, neonatal T cells appear wired to establish a state of tolerance to self and allogenic antigens at baseline homeostatic conditions, while simultaneously being poised to mount a rapid effector response upon infectious challenge or injury. B cell responses in the neonatal period are also distinct from adult. Fetal precursor cells preferentially give rise to B1-B cells that persist in the developing spleen at least 4 weeks after birth. These cells recognize self and microbial antigens and secrete low-affinity antibodies with broad specificities.^132^ The newborn B cell compartment primarily consists of naïve and transitional B cells and few memory cells.^133,134^

*Suppressor cells*: Specialized populations of suppressor cells are found in the early life period that control inflammation and also contribute to age-specific susceptibility to a pathogen and disease. IL-10 producing regulatory B cells are prevalent in human neonates and play a role in RSV clearance.^135^ Myeloid-derived suppressor cells are a heterogeneous population of granulocytic or monocytic cells that suppress innate and adaptive immune responses and play a role in establishing fetal tolerance *in utero*.^136,137^ CD71^+^ erythroid cells are enriched in the spleen of neonatal mice and in human cord blood and suppress both myeloid cell responses and T cell activation through their production of arginase 2.^138^

## Microbial education of perinatal immunity

Unique forces drive early life immunity, and a microbial imprint of this system during the ‘window of opportunity’ may take the form of programming of either the development of immune cell subsets themselves or their functionality. Favorable microbial exposures could direct hematopoietic progenitors down immune developmental pathways that serve unique early life milieus. An alteration in their homeostasis and ability to precisely differentiate may result in losses in immune lineages, or conversely, may cause an increased persistence of fetal-derived immune cell subsets, both scenarios that would impact long-term immune repertoires. Encounters with microbes and microbial components in early life may also program the function of immune cells, some of which take up long-term residence in tissues, to influence host physiological processes and protect from disease throughout life. When and how microbial inputs are interpreted in these distinct early life environments may alter the nature of developmental immune layering, impact long-term immune function, and drive hypersensitivity.

Microbial inputs may originate either from endogenous compounds that are synthesized by microbes such as lipopolysaccharide (LPS) and flagellin or by dietary components that are metabolized by the microbiota, for example, SCFAs produced from dietary fiber. Broad manipulations of the microbiome with antibiotic depletion or germfree rearing have provided important insights into the contributions of the early life microbiome on immune development and later life disease susceptibility. However, microbiome interventions applied maternally can alter both the maternal and offspring microbiomes, rendering it difficult to distinguish gestational from neonatal influences on offspring immune outcomes. Identifying mechanisms by which specific microbes and microbial products affect offspring immune phenotypes may alleviate these concerns and numerous reductionist approaches in animal models have been employed to identify such causal relationships. These primarily involve either starting with an immune phenotype and narrowing down the microbiota to identify specific bacteria that influence the phenotype, or starting with bacteria-derived compounds and metabolites, and seeking to understand their effect on the immune system. A discussion of some of these studies follows.

### Perinatal antibiotics and germfree animals

Epidemiologic and experimental data support a major impact of perinatal antibiotics on numerous aspects of immune system development and function that modulate susceptibility to inflammatory diseases in offspring. Antibiotic exposure *in utero* was positively associated with atopic dermatitis, food allergies and very-early onset IBD within the first year of life.^139,140^ Similarly, antepartum antibiotic exposure of pregnant C57BL/6 mice and colitis-prone IL-10-deficient mice led to altered intestinal bacterial colonization in offspring and increased susceptibility to DSS colitis in adulthood.^141,142^ We have shown that antibiotics exposure of pregnant C57BL/6 dams impacts the development of innate-like lymphocytes in the thymus of offspring that alters susceptibility to experimental colitis in later life.^143^ TF PLZF expressing thymic cells did not develop efficiently in mice that were treated with broad-spectrum antibiotics in the perinatal period, but mice treated in later life were spared. Microbe-induced altered development of thymic innate and innate-like cells in early life persisted into adulthood and contributed to increased susceptibility to DSS-induced colitis that could be reversed by the transfer of PLZF^+^ cells from mice that developed with normal microbiota in early life. Exposure of pregnant C57BL/6 mice to a cocktail of antibiotics decreased the numbers of circulating bone marrow neutrophils and granulocyte/macrophage-restricted progenitor cells (GMPs) in offspring that was associated with increased susceptibility to *E. coli* K1 and *Klebsiella pneumoniae* sepsis.^89^ The balance between Th17 and Treg cells, both important players in mucosal homeostasis,^144^ was also affected by antibiotics exposure of murine dams. There were reduced numbers of IL-17 producing cells in the intestinal lamina propria of offspring of antibiotic treated murine dams.^89^ In the non-obese diabetic (NOD) mouse model, antibiotics treatment of pregnant NOD mice also reduced the numbers of IL-17 producing CD4^+^ and CD8^+^ T cells in the mesenteric lymph nodes and Peyer’s patches with a concomitant increase in FOXP3^+^ Treg cells, that protected offspring from T1D.^145–147^ Interestingly, however, vancomycin treatment of C57BL/6 breeders reduced colonic Treg cells in offspring and increased their susceptibility to asthma.^148^ Impaired anti-viral responses and deficient production of IFNγ by CD8^+^ T cells were observed in neonatal offspring of antibiotic-treated murine dams.^149^ However, antibiotic treatment in the neonatal period lead to dysregulated vaccine responses with decreased IgG production and increased IFNγ production by T cells in adult mice.^150^

Similar to offspring of antibiotic treated murine dams, reduction in GMPs and lower plasma G-CSF levels were also observed in neonatal GF mice, suggesting that the immune impairments were due to alterations in the perinatal microbiome rather than confounding off-target effects of antibiotics.^89^ In GF mice, an early life defect in differentiation potential and self-maintenance capacity of bone marrow GMPs persisted into adulthood and could be restored partially by re-introduction of commensal microbes.^151^ GF mice also displayed increased serum IgE levels that were only normalized by conventionalization with normal microbiota before 4 weeks of age.^152^ Neonatal GF Swiss Webster (SW) mice but not GF C57BL/6 mice had higher numbers of iNKT cells in the colonic lamina propria, that increased their susceptibility in models of oxazolone-induced colitis and ovalbumin-induced allergic asthma.^153^ Elevations in colonic iNKT cells in GF mice were abrogated by re-introduction of conventional microbes at birth but not in adulthood, revealing an early life period during which the gut microbiota affects intestinal iNKT cell numbers. Early life microbes also regulate intestinal transcriptional programs, and alterations in expression of immune-related genes have been documented in GF mice that were only restored by colonization at birth but not in adulthood.^154^ Maternal microbes also imprint distinct patterns of gene expression in small intestinal mucosa of offspring in a mouse model of gestation-only microbial colonization.^39^

### Endogenous microbial compounds

Endogenous microbial compounds can penetrate into the adult murine host, especially from bacteria in the lower small intestine.^155^ Isotope-labeling studies show the presence of microbial molecules of maternal origin in the placenta and the developing fetus as well as in breastmilk that drive the development of innate immune system and maturation of the intestinal epithelium in murine offspring.^39^ Microbial ligands for the aryl hydrocarbon receptor, which is expressed in the placenta, liver, and mucus membranes, promoted the development of intestinal NKp46^+^ ILC3s in the offspring. Components of bacterial cell wall and capsule are recognized by innate pattern recognition receptors expressed on a plethora of host cells in the intestines. Lipopolysaccharide (LPS) could restore impaired anti-viral CD8^+^ T cell responses in neonatal offspring of antibiotic-treated murine dams,^149^ suggesting that endogenous LPS from the neonatal microbiome might be required for proper CD8^+^ T cell function.^156^ LPS and microbial ligands for other PRRs have been shown to have diverse effect on central nervous system development and function.^157^ We showed that capsular polysaccharide A (PSA) from *Bacteroides fragilis* promoted the development of PLZF^+^ innate-like lymphocytes in the thymus of neonatal mice in a TLR2 dependent manner.^143^ *B. fragilis* sphingolipid also inhibited iNKT cell proliferation in the neonatal colon that protected from colitis in later life.^158^ Flagellin is a ligand for TLR5 that is expressed on intestinal epithelial cells primarily in early life.^159^ Neonatal flagellin and TLR5 interactions influence the composition of the murine microbiota throughout life and impact susceptibility to metabolic syndrome.^160^ In mice transgenic for a T cell receptor recognizing Lachnospiracea flagellin, antigen-specific RORγt^+^ Treg cells were generated in the early life period, specifically during the weaning process, and protected them from severe colitis later in life.^161^ In another TCR transgenic mouse model, recognition of a *Staphylococcus epidermidis* antigen to generate antigen-specific Treg cells also occurred during the second week of birth and was necessary to maintain tolerance to this antigen in adulthood.^162^ There is still a dearth of information on effector microbial products and further research is needed to understand the nature of specific microbial molecules that elicit immune modulatory effects in early life.

### Diet and dietary components metabolized by microbiota

In animal models, maternal consumption of a high-fat diet (HFD) altered the microbiota and the nature of microbial exposures in both pregnant mothers and their offspring.^163–165^ Maternal obesity in murine dams fed a chronic HFD correlated with restricted expansion of hematopoietic stem and progenitor cells (HSPCs) in the fetal liver of offspring and altered lineage specification that resulted in distinct patterns of reconstitution upon adoptive transfer.^166^ Children with acute malnutrition also have impaired development of their gut microbiota (.^167^ Undernutrition in early life is associated with a number of adverse outcomes including immune dysfunction.^168^ IgA responses against specific bacterial taxa was altered in undernourished children that disrupted normal postnatal assembly of the microbiome and led to an enrichment of disease-promoting microbial communities including those containing members of Enterobacteriaceae and associated enteropathogenic strains.^169^ Further, transplantation of this bacterial consortium from undernourished children into gnotobiotic recipient altered gut barrier function and transmitted the weight loss phenotype.^169^ Dietary components may also be metabolized by the maternal and postnatal microbiota to influence immunity.^170,171^ It is estimated that there are 500–1000 microbial operating taxonomic units in the mammalian gut, giving the microbiome as a whole, an immense capacity to code for nutrient transporters and metabolizing enzymes.^172^ Maternal microbes can metabolize SCFAs by the fermentation of dietary fibers. SCFAs imprint the capacity to generate regulatory T cells in the fetal lung that protects adult murine offspring from house dust-mite induced allergy airway disease.^40^ Maternal supplementation with the SCFA acetate during pregnancy was also shown to rescue thymic and T cell developmental defects by upregulating AIRE (autoimmune regulator) expression and enhancing Treg cell generation in a mouse model of pre-eclampsia.^173^ Retinoic acid (RA) is a Vitamin A metabolite whose synthesis by intestinal epithelial cells is regulated by commensal bacteria.^174^ Maternal RA was shown to regulate CXCL13 production by murine fetal mesenchymal cells and the differentiation of lymphoid tissue inducer (LTi) cells.^175^ Vitamin B2/riboflavin metabolites produced by the microbiota before weaning promoted the development of MAIT cells in the murine thymus.^176^

## Practical implications of the early life window of opportunity

It is evident that the immune system is amenable to beneficial programming by microbes in the perinatal period. Lack of appropriate microbial exposures, either due to microbial perturbation during pregnancy, antibiotics use in the perinatal period, malnutrition, or exposure to low diversity microbiota can lead to pathological imprinting that contributes to increased susceptibility to asthma, allergies, and other chronic inflammatory conditions.^177,178^ A practical approach to prevent such pathologies is to identify components of the microbiota that may be used clinically to ensure healthy imprinting in early life. A combination of probiotics, prebiotics, and immunomodulatory metabolites specific to the early life period may then be designed that promote favorable immune outcomes and decrease the incidence of inflammatory pathologies in later life. These may be administered to mothers during pregnancy, to mother-infant dyads postpartum, or directly to the infant.^179^ Another strategy is the transfer of mother’s vaginal and fecal microbiota at birth to introduce pioneer bacteria that build a robust microbial community that promotes offspring health. While antibiotic treatment during the neonatal period is causally linked to pathological imprinting, it is difficult to envision how one might avoid these treatments given their critical role in clearing infections in infants. Similarly, consumption of high-fat diets by mothers and weanlings can increase susceptibility to metabolic disorders; however, such high-calorie foods are crucial for meeting nutritional needs in developing countries. One strategy might be to prevent microbial alterations brought about by these treatments and behaviors with dietary prebiotics and probiotics.

Commonly administered probiotics belong to the genera *Bifidobacterium* and *Lactobacillus* and their use may prevent antibiotic-induced diarrhea and reduce the risk of NEC in preterm infants.^180^ However, no beneficial effect of probiotics in acute gastroenteritis in children was found.^181^ *Bifidobacterium longum* subsp. *infantis* EVC001 is a gut symbiont that can efficiently utilize human milk oligosaccharides (HMOs) into organic acids that lowers intestinal pH and can decrease markers of enteric inflammation in infants.^182^ In neonatal mice, colonization with *Lactobacillus rhamnosus* GG enhanced intestinal functional maturation and IgA production that protected from inflammation in later life.^183^ Probiotics may modulate the composition of the gut microbiota, enhance epithelial barrier function, and direct antagonism against pathogens. However, the precise mechanism of their action remains to be determined and is likely strain dependent. Prebiotics are substrates that are selectively utilized by host bacteria to confer a health benefit. Dietary fiber that is metabolized to SCFAs (acetate, propionate, butyrate) by gut microbes has been shown to influence microbial composition and the generation of immune cell subsets.^184^ Prebiotics such as short-chain galactooligosaccharides and long-chain fructooligosaccharides are routinely added to infant formula and have been shown to increase the abundance of *Bifidobacteriu*m.^185^ However, further studies are needed to determine their impact on immune function and the composition of the microbiota in early and later life. Crucial information is also needed on the possible long-term effects of early life pro- and pre-biotic administration on immune function and disease susceptibility. Probiotics in infant formula can deliver a large bacterial inoculum into a still-developing intestinal environment. Alterations in intestinal epithelial gene expression profiles, immune cell distribution, and function as well as the microbial landscape could potentially persist into adulthood leading to unwanted long-term health consequences.

Efficacy of vaccine responses can also be potentially programmed by intestinal microbes that may act as immunomodulators as well as natural adjuvants. Low antigen-specific plasma cell frequencies and IgG levels were observed in germfree and antibiotic-treated *Tlr5^−/-^* mice upon vaccination with a trivalent inactivated influenza vaccine, that was restored by reestablishment of the microbiota.^186^ Similarly, intestinal microbiota and recognition of the Nod2 sensor were required for inducing an immune response to cholera toxin in mice.^187^ In humans, variations in microbial communities may provide one explanation for the poor response to oral vaccines in low-income countries. While a causal link between specific community composition and vaccine responsiveness still needs to be established, some bacterial species have emerged as modulators of humoral and cellular immunity in responders. A robust IgA response to oral rotavirus vaccine in Ghanaian infants correlated with an increased relative abundance of stool *Streptococcus bovis* and a decreased abundance of Bacteroidetes.^188^ A higher polio-specific T cell response and serum IgG levels in Bangladeshi infants that received oral polio vaccine correlated with a higher abundance of *Bifidobacteria* and lower *Pseudomonadale*s.^189^ Approaches to test the immunomodulatory and adjuvant effects of probiotics on beneficial vaccine responses need to be defined.^190^ As with any probiotic use in early life, choice of strain and dose and timing of administration must carefully be evaluated along with long-term health consequences.

## Conclusion

**Figure 3.**
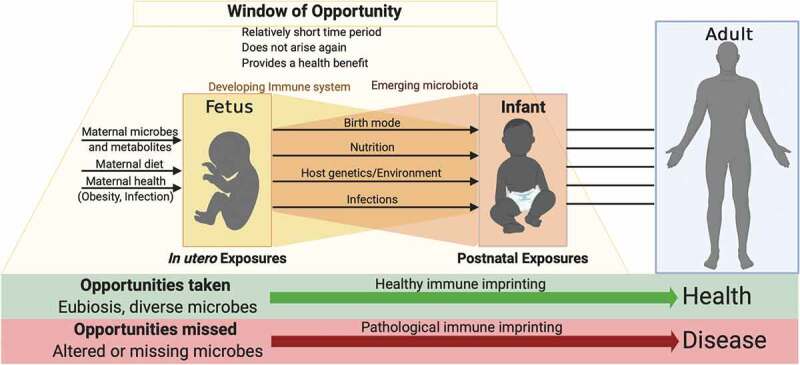
**Of opportunities missed and taken**. Early life microbial exposures influence developmental imprinting of the immune system that determines health and disease susceptibility in later life. Favorable imprinting of developing immunity by microbes may occur during an early life time window of opportunity. This window of opportunity, by definition, is predicted to be relatively short, to not arise again in later life and must provide a health benefit to the offspring. Numerous factors during pregnancy and in the postnatal period influence the quality of microbial exposures that have the potential to imprint immune functionality. Treatments and behaviors that disrupt these exposures may lead to pathological imprinting, altering risk for disease throughout life.

In summary, the maternal and neonatal microbiomes are important regulators of health and influence the development of the early life immune system. The precise time frame for this ‘window of opportunity’ when immune cells are likely to be malleable to microbial influences is as yet undetermined. This period likely spans the time from fetal development to weaning or introduction of solid foods (Figure 3). Specific microbial encounters in the first 100 days of life were shown to influence the incidence of asthma in high-risk infants.^177^ In mice, a ‘weaning reaction’ encompassing the first 2–4 weeks of life was demonstrated to be critical for mitigating disease risk in adulthood.^191^ Distinct immune cell subsets are generated from precursor cells arising sequentially from the yolk sac, fetal liver, and bone marrow and may be subject to imprinting by specific exposures arising during these times. Animal models have been vital in providing insights into the perinatal development of the immune system. However, developmental timelines differ between species as do the quality of microbial exposures in the perinatal period. The influence of *in utero* microbial exposures on human fetal immune development and hematopoiesis remains largely unknown. Most studies have utilized cord blood at the time of parturition to make inferences on the earliest immune cells. However, human cord blood measurements are not representative of postnatal immunity, and display diverse immune phenotypes that appear to converge in the first few weeks of life.^52^ Further, while cell subsets such as B, NK, and DCs reach adult-like phenotypes during the first 3 months of life, T cell phenotypes do not converge during this period, suggesting that specific cell populations have different periods of calibration and time frames when they are amenable to microbial imprinting.^52^ Recent single-cell multi-omics technologies utilizing small numbers of cells from human fetal tissue have provided a new-detailed view of the complex immune development and structural and cellular communications occurring over ontogeny.^82,192^ More studies are needed to precisely define the temporal contributions of specific microbial species of maternal and neonatal origin on immune cells and their function.

Reductionist approaches in animal models have been used to delineate causal relationships between early life microbes and disease susceptibility. However, while these studies have been and will continue to be critical for understanding basic mechanisms of early life host-microbiome interactions, how they may be translated to clinical applications and the development of microbiome based therapeutics will be key. Efforts to better model clinically relevant microbiome perturbations will be important as microbiome-based therapeutic strategies are developed. Applying pharmacologically relevant doses of antibiotics to animals, evaluating their off-target effects, and elucidating how microbiome perturbations might impact developmental processes will be important to evaluate safety, efficacy, and promise of such endeavors. Deeper mechanistic and functional insights into microbe-immune interactions at specific time periods are thus critical to inform disease mitigating strategies that modulate early life immune imprinting.
